# Non-empirical atomistic dipole-interaction-model for quantum plasmon simulation of nanoparticles

**DOI:** 10.1038/s41598-017-16053-6

**Published:** 2017-11-17

**Authors:** Jaechang Lim, Sungwoo Kang, Jaewook Kim, Woo Youn Kim, Seol Ryu

**Affiliations:** 1 KAIST, Department of Chemistry, 291 Daehak-ro, Yuseong-gu, Daejeon 34141 Republic of Korea; 2 Chosun University, Department of Chemistry, 309 Pilmun-daero, Dong-gu, Gwangju 61452 Republic of Korea

## Abstract

Plasmonic nanoparticles in the quantum regime exhibit characteristic optical properties that cannot be described by classical theories. Time-dependent density functional theory (TDDFT) is rising as a versatile tool for study on such systems, but its application has been limited to very small clusters due to rapidly growing computational costs. We propose an atomistic dipole-interaction-model for quantum plasmon simulations as a practical alternative. Namely the atomic dipole approximation represents induced dipoles with atomic polarizabilities obtained from TDDFT without empirical parameters. It showed very good agreement with TDDFT for plasmonic spectra of small silver clusters at much lower computational cost, though it is not appropriate for molecular-like excitations. It could also reproduce the plasmonic band shift experimentally observed in sub-10 nm silver particles.

## Introduction

Noble metal nanoparticles of which sizes are comparable to light wavelengths show characteristic colors due to the strong absorption/scattering of light in the visible region. Electromagnetic waves induce the collective oscillation of free electrons in the particles, which is referred to as localized surface plasmon resonance (LSPR). Such an intriguing feature can be tuned by the size, shape, materials, and surrounding environments of nanoparticles for various applications in chemistry, physics, and biomedical fields^1–4^. Therefore, understanding of the structure-optical property relation is of importance for engineering. Extensive experiments have been performed for that purpose, but they mostly focused on particles with diameter above 10 nm^5–7^. Accordingly, theoretical methods in the classical regime have been well established for the large nanoparticles^8^.

Nanoparticles below 10 nm have extremely high surface-to-volume ratios. Thus, the contribution of surface atoms to light scattering becomes severe, giving rise to substantially different optical properties from those of larger nanoparticles with strong dependence on atomistic structure. For example, it has been observed that the LSPR band of nanoparticles gradually blue-shifts as their size goes down below 10 nm^9–12^. Classical theories cannot be applied to such quantum-sized particles. A quantum-corrected model of the classical scattering theory has been proposed^13,14^. Alternatively, time-dependent density functional theory (TDDFT) became popular thanks to its reasonable accuracy and efficiency^15–22^. At present, however, its application has been limited to very small particles with less than a few hundreds of atoms due to rapidly growing computational costs. Acceleration of TDDFT is feasible by employing a real-time propagation method^22,23^, the Liouville-Lanczos approach^24^, and the complex polarizability algorithm^25,26^. Nonetheless, it is expected that there is a technical barrier to deal with more than a few thousands atoms. Therefore, a new efficient method bridging the classical and quantum regimes is necessary.

Among classical methods, discrete dipole approximation (DDA)^27,28^ and its variants^29^ have been extensively used to study plasmonic nanoparticles due to a facile analysis of calculation results. However, the fact that DDA typically adopts bulk dielectric constants hinders its application to quantum-sized nanoparticles. Recently, Jensen and coworkers developed an atomistic version of DDA, called the coordination-dependent discrete interaction model (cd-DIM)^13^. It considers different local environments of each atom on the surface by introducing coordination-dependent dielectric constants. As a result, it could effectively describe the blue shifting trend of LSPR bands.

The success of cd-DIM attributes from introducing a tunable plasmon frequency that is parameterized with an empirical value for surface plasmon together with an experimental bulk value under the Drude-model approximation. However, such an empirical value is not available for most plasmonic materials. In this regard, we propose a non-empirical atomistic dipole-interaction model for quantum plasmon simulations. Namely our atomic dipole approximation (ADA) also uses atomic dipoles, but atomic polarizabilities can be obtained solely from a first principles theory. Therefore, it is feasible to apply it to systems for which empirical parameters are not readily available. We show that ADA quantitatively reproduces the TDDFT spectra of small silver clusters. It could also naturally illustrate the size-dependent plasmon shift, indicating the feasibility of its application to quantum plasmonics.

## Theoretical Methods

In general, the polarizability of a discretized medium is proportional to its volume. The polarizability of the *I*-th atom in a nanoparticle (*α*_*I*_) can be approximated by multiplying a relative volume ratio to the polarizability of the corresponding free atom (${\alpha }_{{\rm{I}}}^{{\rm{free}}}$), where the ratio is given by the effective volume (${V}^{{\rm{eff}}}$) of the *I*-th atom divided by the volume of the free atom (${V}^{{\rm{free}}}$) as follows:

1 $${\alpha }_{I}=\frac{{V}^{{\rm{eff}}}}{{V}^{{\rm{free}}}}{\alpha }_{I}^{{\rm{free}}}=\frac{{V}^{{\rm{eff}}}}{{V}^{{\rm{free}}}}\frac{{e}^{2}}{m}\sum \frac{{f}_{n}}{{\omega }_{n}^{2}-{\omega }^{2}-i\omega \delta },$$

where *e* and *m* are the electron charge and mass, and ${\omega }_{n}$, ${f}_{n}$, and $\delta$ are the *n*-th absorption frequency, its oscillating strength and damping factor, respectively. ${\omega }_{n}$ and ${f}_{n}$ of a free atom can directly be obtained from TDDFT. We assumed that $\delta$ depends on the coordination factor, *X*, of each atom in a nanoparticle, which is given by

2 $$\delta (X)=\mathrm{(1}-X){\delta }_{1}+X{\delta }_{2},$$

where *X* is defined as^30^

3 $$X=\frac{{\rm{\min }}\,(C{N}_{I},C{N}_{{\rm{\max }}})}{C{N}_{{\rm{\max }}}}\mathrm{.}$$

**Figure 1:**
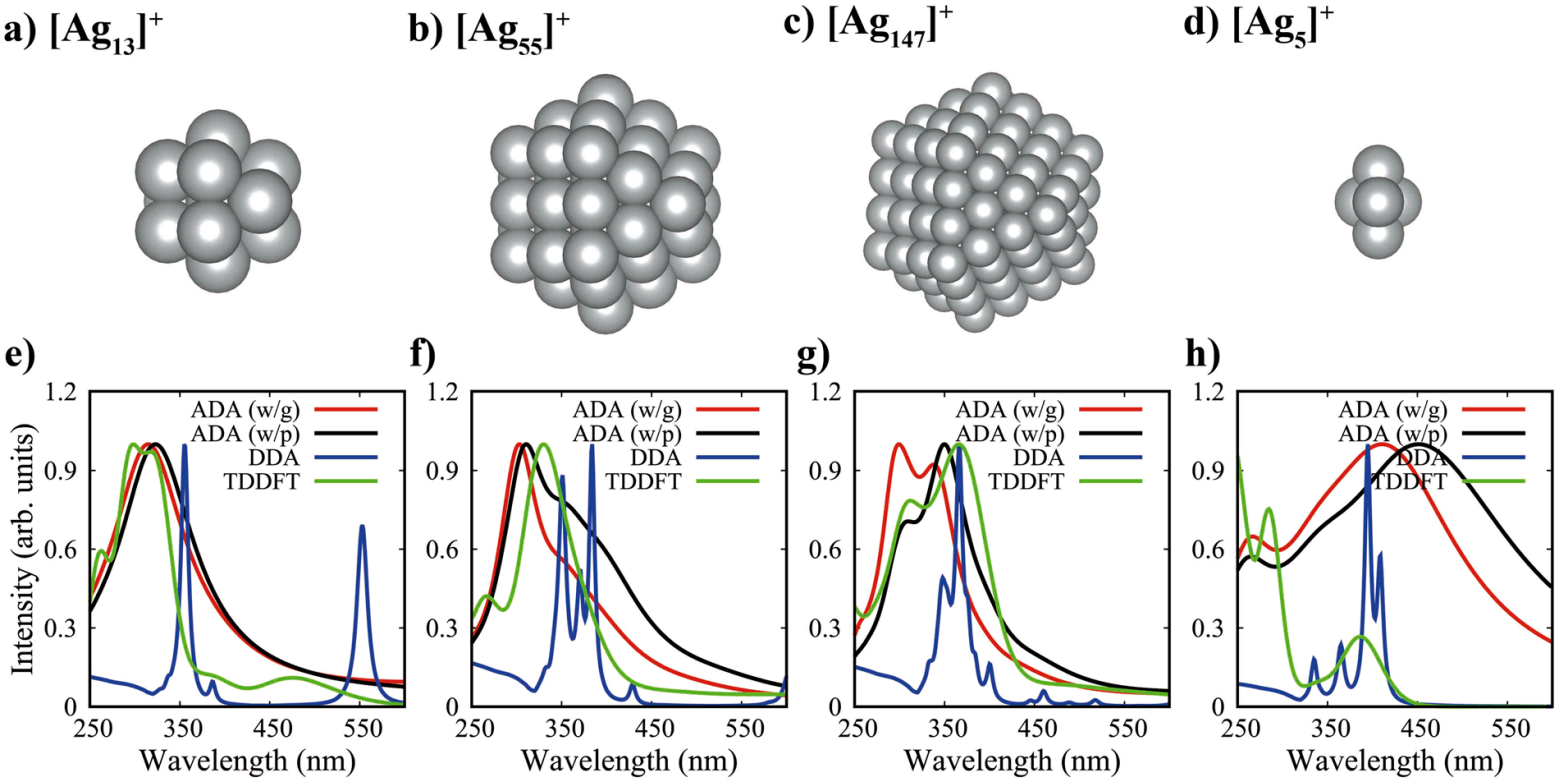
Small silver nanoclusters with the cuboctahedron symmetry [(**a**,**b**,**c**, and **d**)] and their optical spectra calculated through ADA, DDA and TDDFT [(**e**,**f**,**g**, and **h**)], respectively. ADA (w/g) and (w/p) denote spectra from simulations with the Gaussian and point charges, respectively.

Here, $C{N}_{I}$ and $C{N}_{{\rm{\max }}}$ denote the coordination number of atom *I* and the maximum coordination number of atoms in the particle, respectively. The damping parameters ${\delta }_{1}$ and ${\delta }_{2}$ were determined for ADA spectra to be the best fit to TDDFT results in Fig. 1 (for details in Supplementary Information). ${V}^{{\rm{eff}}}$ is calculated through the Hirshfeld partitioning of the total electron density of a nanoparticle given by DFT^31^. If a nanoparticle is too large to be computed by DFT, it can be replaced by a reference one that is small enough for DFT calculations. Then, the effective volumes of atoms being in a similar environment to original ones are taken. In this case, the similarity between two atoms is determined by comparing their coordination numbers as suggested in ref.^30^.

The atomic polarizabilities obtained in such a way are embedded in each atomic position of a nanoparticle. Subsequently, the total interaction energy between induced dipoles is minimized using a self-consistent screening (SCS) method as in DDA^29^, which can be written as

4 $${\alpha }_{I}^{{\rm{SCS}}}={\alpha }_{I}+{\alpha }_{I}\sum _{I\ne J}^{N}{T}_{IJ}{\alpha }_{J}^{{\rm{SCS}}},$$

where ${T}_{IJ}$ is the interaction tensor between dipoles *I* and *J*. In our approach, atomic dipoles are approximated as a polarizable sphere with Gaussian charge distribution whose width is given by

5 $${R}_{I}={(\sqrt{\frac{2}{\pi }}\frac{{\alpha }_{I}}{3})}^{\mathrm{1/3}}\mathrm{.}$$

In fact, the same idea has successfully been used to obtain van-der-Waals coefficients^32,33^. Finally, the optical absorbance of the nanoparticle is obtained from the total polarizability:

6 $${\sigma }_{{\rm{abs}}}(\omega )=\frac{4\pi \omega }{c}{\rm{Im}}\{{\alpha }_{{\rm{total}}}(\omega )\}=\frac{4\pi \omega }{c}{\rm{Im}}\{\sum _{I}^{N}{\alpha }_{I}^{{\rm{SCS}}}(\omega )\}\mathrm{.}$$

The aforementioned scheme of ADA and the conventional DDA have been implemented in our code, ACE-Molecule, which is a general purpose quantum chemistry package based on a real space grid method^34,35^. In DDA simulations, discrete elements were scaled down to atom size, and the corresponding polarizabilities were derived from the experimental dielectric function of bulk silver^36^ according to the Clausius-Mossotti and lattice dispersion relations^27^. All DFT and TDDFT calculations were also performed using ACE-Molecule. The simulation box for DFT and TDDFT was made by the superposition of spheres with radius of 3.4 Å centered on each atom of a particle. The grid spacing of 0.212 Å was employed. The PBE functional^37^ and the norm-conserving pseudopotential of Ag^38^ were used for all calculations. We considered sphere-like nanoparticles of cuboctahedron symmetry. Their atomistic structures were generated by adding Mackay layers one-by-one to a central atom with the bond length of 2.89 Å. For the sake of computational efficiency, we removed one electron from each particle so as to make closed shell electronic structures.

The datasets generated during and/or analysed during the current study are included in this published article and its Supplementary Information file.

## Results and Discussion

Figure 1 shows the computed optical spectra of ${{\rm{Ag}}}_{5}^{+}$, ${{\rm{Ag}}}_{13}^{+}$, ${{\rm{Ag}}}_{55}^{+}$, and ${{\rm{Ag}}}_{147}^{+}$. First of all, the ADA results for ${{\rm{Ag}}}_{13}^{+}$, ${{\rm{Ag}}}_{55}^{+}$, and ${{\rm{Ag}}}_{147}^{+}$ [ADA(w/g)] are very close to those of TDDFT except a little blue shift, whereas DDA gives completely different results. The good agreement between ADA and TDDFT is partly because the atomic polarizabilities in ADA have been obtained from same TDDFT calculations, while the bulk parameters used in DDA were obtained from experimental data^36^. To further understand the notable accuracy of ADA, we investigated the effects of the Gaussian charge embedding and the coordination-dependent effective atomic volume. The latter is negligible because the maximum difference between effective atomic volumes is about 0.6%. The Gaussian charge embedding gives rise to a slight hypsochromic change in the spectral feature due to the screening effect on the interaction between induced dipoles at short range. In fact, the use of point charges induces the red shift of the spectra, resulting in slightly better results for ${{\rm{Ag}}}_{55}^{+}$ and ${{\rm{Ag}}}_{147}^{+}$ as shown in Fig. 1 [ADA(w/p)]. The spikey peaks of DDA results roughly represent non-equivalent surface atoms, and the characters of surface atoms tend to converge as the particle size increases. For instance, the twelve atoms of ${{\rm{Ag}}}_{13}^{+}$ are at the surface with the 8:4 ratio of two kinds. Each type would have a unique resonance frequency for the oscillation of induced dipoles, resulting in two separate peaks. Then, the two peaks gradually merge to a single peak for a larger particle, e.g., ${{\rm{Ag}}}_{147}^{+}$. For particles above 10 nm, almost continuous resonance modes are allowed in a certain range of frequency, leading to a broad peak. The same rationale applies to the ADA results, but the damping factors in Eq. (1) which are absent in DDA caused substantial broadening of the peaks as in TDDFT.

**Figure 2:**
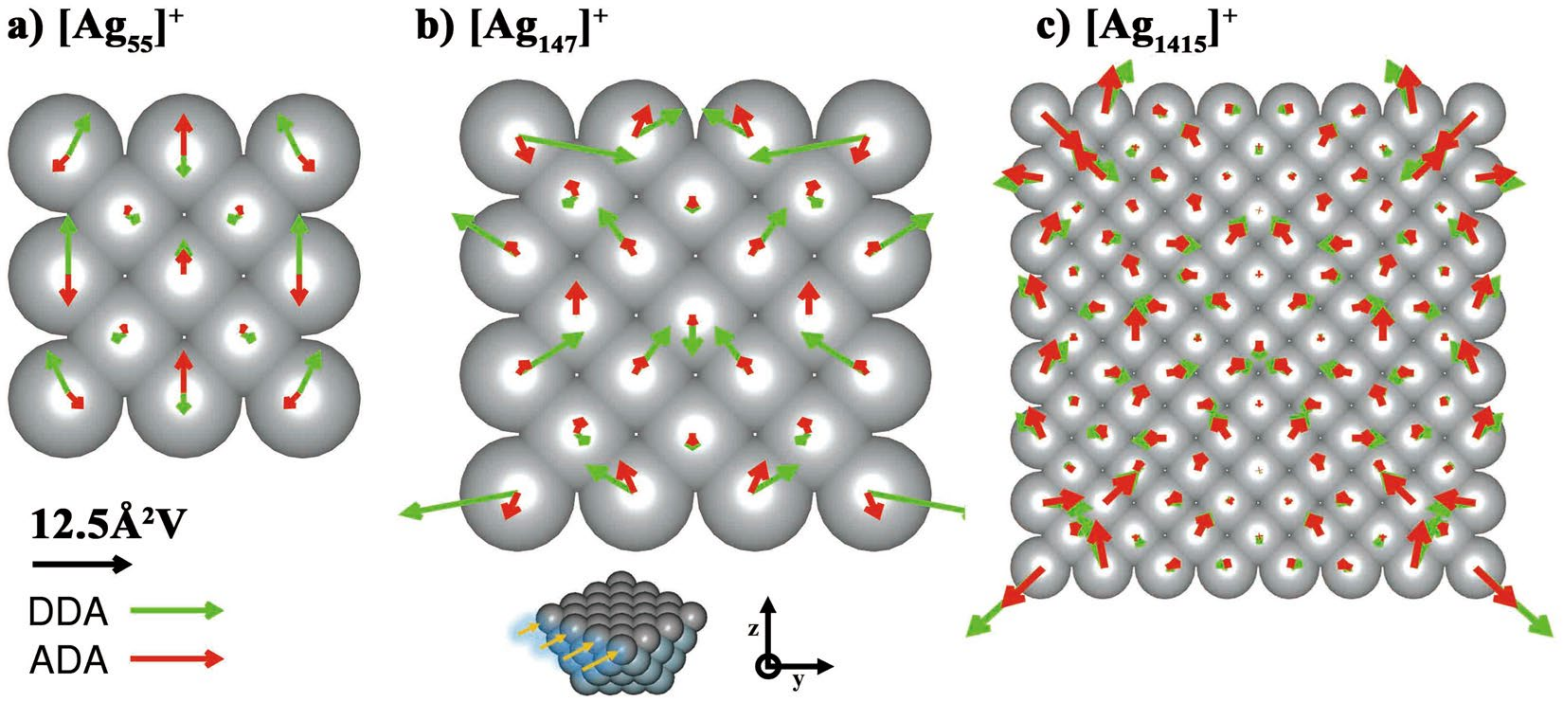
Atomic polarizations of ${{\rm{Ag}}}_{55}^{+}$, ${{\rm{Ag}}}_{147}^{+}$, and ${{\rm{Ag}}}_{1415}^{+}$ obtained using ADA(w/g) and DDA under the unit electric field along the y-direction of light propagating in z-direction. The wavelengths of light are selected at maximum-peak positions, i.e., 313, 300, and 325 nm in ADA(w/g) and 384, 366, and 358 nm in DDA for ${{\rm{Ag}}}_{55}^{+}$, ${{\rm{Ag}}}_{147}^{+}$, and ${{\rm{Ag}}}_{1415}^{+}$, respectively. The upper part of ${{\rm{Ag}}}_{147}^{+}$ nanoparticle in the inset is removed for visualization.

However, it is not sufficient to explain the outperformance of ADA against DDA. We compared the self-consistently converged atomic polarizations between ADA(w/g) and DDA at the maximum peak frequency for ${{\rm{Ag}}}_{55}^{+}$, ${{\rm{Ag}}}_{147}^{+}$, and ${{\rm{Ag}}}_{1415}^{+}$ (Fig. 2). Overall, the polarization difference between ADA and DDA is relatively small in the core region, but it becomes larger as approaching to the surface. Also, the polarization of DDA is larger in magnitude than that of ADA especially on the surface. More specifically, for smaller nanoparticles, ADA shows a relatively small difference between polarizations on the core atoms and the outermost surface atoms, but the difference becomes larger and larger as the particle size increases. In other words, atomic polarizations that are initially spread over the entire region in a small particle begin to localize around surface regions as the particle size increases (e.g., ${{\rm{Ag}}}_{1415}^{+}$ with around 4 nm diameter), giving rise to LSPR as a characteristic optical behavior of bulk nanoparticles. In contrast, DDA gives a substantially large difference between polarizations on the core and the surface atoms already from the smallest particle, ${{\rm{Ag}}}_{55}^{+}$. Eventually, however, both ADA and DDA give similar polarizations for larger particles as noted in ${{\rm{Ag}}}_{1415}^{+}$. On one hand, the resonance energies in the atomic polarizabilities are higher than bulk values, and thus it is expected that they are closer to surface plasmon frequency. On the other hand, in small nanoparticles with a high surface-to-volume ratio, all atoms behave like surface atoms to some extent because of a small difference between core and surface atoms. ADA captures such a feature better than DDA. Consequently, the significant improvement of ADA over DDA would mainly come from the use of TDDFT-generated atomic polarizabilities.

It is noteworthy that ADA is a simple model devised specifically for plasmon simulations. Figure 1(h) exhibits substantial differences between ADA and TDDFT of optical spectra for ${{\rm{Ag}}}_{5}^{+}$. This failure manifests a clear limitation of ADA for molecular-like excitations dominant in such a very small cluster. In molecular-like excitations where quantum nature of discretized energy levels is critical, electrons feel a completely different potential due to the substantial change in electron charge distributions upon the excitation from the ground state to an excited state, meaning that characteristics of dipoles (i.e., atomic polarizability) are significantly changed, which is absent in the ADA method using non-adaptive atomic polarizabilities as an input. Therefore, TDDFT would be a better choice for study on very small clusters. However, it is no longer practical for systems with more than a thousand atoms because of unaffordable computational costs. For intraband transitions such as plasmonic excitations, it is reasonable to assume that the change in a potential by electronic excitations is not significant. This allows us to use a classical model to describe plasmon-like transitions.

**Figure 3:**
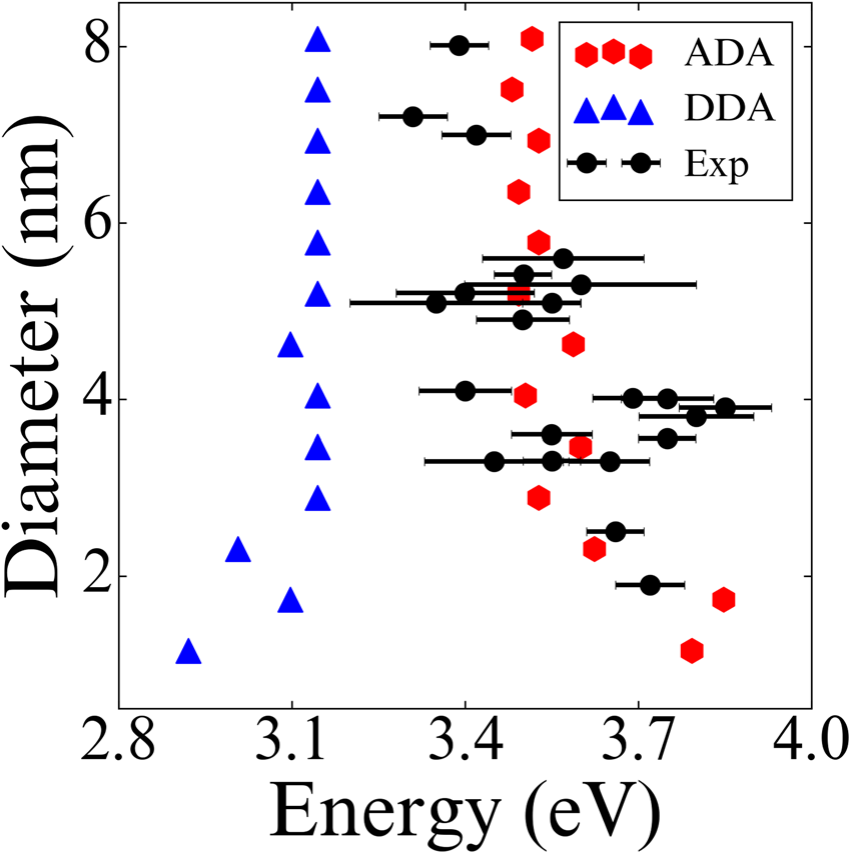
Optical spectra of silver nanoparticles as a function of diameter. (**a** and **b**) are obtained from ADA and DDA, respectively. Both methods used Gaussian charges. The experimental data (the black dots with error bars) is obtained from ref.^12^. The results of ADA and DDA have been red-shifted by 0.3 eV.

To validate ADA for plasmonic nanoparticles with diameter between 3 and 10 nm, we investigated if ADA can reproduce the experimentally observed blue shift of LSPR band as the size of nanoparticles gradually decreases from ~10 nm (12,431 atoms) to ~1 nm (55 atoms). Figure 3 shows the changes in the optical spectra of ADA and DDA for silver nanoparticles as a function of diameter. The results of ADA and DDA have been red-shifted by 0.3 eV for the purpose of comparison, taking into account medium effects in the experimental data^12^. Additionally, the polarizable sphere model with Gaussian charge embedding has been applied to both methods in order to examine the effect solely by the difference in their polarizabilities. ADA well reproduces the blue shift without help from empirical parameters, whereas DDA results are far off the experimental data as reported previously^13^.

It is very interesting to see that, with the particle size decreasing, both theory (ADA and DDA) and experiment start to show quantum size effects and spectral changes around 4 nm. This is also consistent with the change in the distribution of atomic polarizations with respect to the particle size as shown in Fig. 2; as the particle size decreases below 4 nm, the atomic polarizations localized at the surface begin to spread over the core region. A similar interpretation based on TDDFT spectra has been reported in ref.^16^. Since it is known that metal-insulator transition for silver occurs near 4.1 nm^39^, much less than bulk electron mean free path, it is reasonable that more of atomistic characteristics and quantum plasmon effects are revealed in such size range. Regarding optical spectral features of silver nanoparticles, both cd-DIM model^13^ and our ADA method equally well display the size-dependent LSPR blue shifting trend as observed in experiments. It should be noted, however, that the improvement in ADA over cd-DIM lies in the fact that the former need not adopt a tunable plasmon frequency depending on an empirical parameter because it uses atomic polarizabilities generated from a first principles theory.

For computational efficiency, it should be noted that ADA is incomparably faster than TDDFT. In the case of ${{\rm{Ag}}}_{147}^{+}$, for example, computational time of TDDFT was about two days on 128 CPU cores, whereas that of ADA was only around a few minutes even on a single CPU core. For a medium sized nanoparticle with 6 nm diameter (3,871 Ag atoms) to which TDDFT cannot be applied, ADA calculations took about 150 seconds on 48 CPU cores.

## Conclusions

In order to calculate optical spectra for small metal nanoparticles in the quantum regime, we developed ADA using atomic polarizabilities obtained from TDDFT. As an atomistic reformulation of DDA without introducing empirical parameters, ADA could quantitatively illustrate the characteristic size-dependent plasmon blue-shifts of sub-10 nm silver nanoparticles observed in experiments. We believe that the ADA method can be a competitive and practical alternative to DDA and TDDFT for quantum plasmon simulations, in which bulk dielectric functions for common DDA simulations are simply wrong, but TDDFT cannot be applied due to too high computational costs. The non-empirical nature of ADA will allow for its application to a variety of systems for which empirical parameters are not readily available such as small alloy nanoparticles as well as quantum-sized particles.

## Electronic supplementary material

Supplementary Information
