# Supplementary material for: Non-empirical atomistic dipole-interaction-model for quantum plasmon simulation of nanoparticles
Source: Sci Rep. 2017 Nov 17;7:15775. doi: 10.1038/s41598-017-16053-6 (PMC5693991; doi:10.1038/s41598-017-16053-6)
Supplement: Supplementary file 1 — Supplementary Information [file 41598_2017_16053_MOESM1_ESM.doc]

Supplementary Information for

Non-empirical atomistic dipole-interaction-model for quantum plasmon simulation of nanoparticles

*Jaechang Lim,^1^ Sungwoo Kang,^1^ Jaewook Kim,^1^ Woo Youn Kim,^1^* and Seol Ryu^2^**

^1^Department of Chemistry, KAIST, 291 Daehak-ro, Yuseong-gu, Daejeon 34141, Republic of Korea

^2^Department of Chemistry, Chosun University, 309 Pilmun-daero, Dong-gu, Gwangju 61452, Republic of Korea

**Corresponding Author**

*E-mail: sryu@chosun.ac.kr (S.R.)., *E-mail: wooyoun@kaist.ac.kr (W.Y.K.).

**1. Atomic polarizability of silver**

An atomic polarizability can be written as follow:

, (S)

where excitation energy ($\omega_{n}$) and oscillator strength ($f_{n}$) were obtained using time-dependent density functional theory implemented in Gaussian09^1^ (Table S1). We assumed that the damping factor ($\delta$) depends on the coordination factor, X, of each atom in a nanoparticle, which is given by

$\delta(X)=\delta_{1}(1-X)+X\delta_{2}$. (S)

where *X* is defined as^2^

$X=\frac{MIN(CN_{I},CN_{\max})}{CN_{\max}}$ (S)

with $CN_{I}=\sum_{J\neq I}^{I} f_{IJ}$ (S)

and

$f_{IJ}=\left\{\begin{matrix} 1 \\ \frac{1}{2}\left(1+\cos\left(\frac{\pi\left|r_{IJ}\right|}{\left|R_{\max}-R_{\min}\right|}\right)\right) \\ 0 \end{matrix}\right.$ $\begin{matrix} r_{IJ}<R_{\min} \\ R_{\min}<r_{IJ}<R_{\max} \\ r_{IJ}>R_{\max} \end{matrix}$ . (S)

Here, r_IJ_ is the distance between atoms *I* and *J*, and *R*_min_ and *R*_max_ were taken from ref 2. In eq S2, *δ*_1_ and *δ*_2_ were determined for ADA spectra to be the best fit to TDDFT results in Figure 1 of the main text, resulting in 2.48 eV and 0.31 eV, respectively.

**
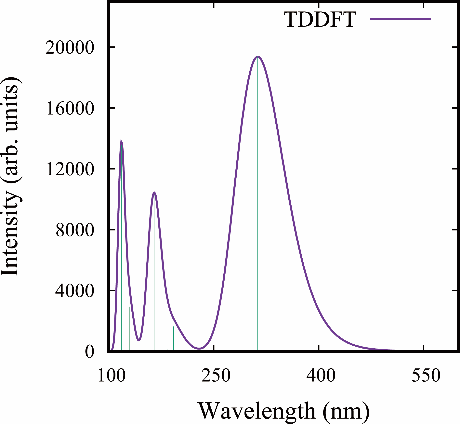
**

**Figure S1.** Absorption spectrum of a silver atom obtained from TDDFT with the PBE functional and Lanl2tz^3^ basis set.

| Excitation energy (eV) | Oscillator strength |
| --- | --- |
| 3.961 | 0.2451 |
| 3.961 | 0.2451 |
| 3.961 | 0.2451 |
| 6.4402 | 0.0205 |
| 6.4402 | 0.0205 |
| 6.4402 | 0.0205 |
| 6.7215 | 0.0012 |
| 6.7215 | 0.0012 |
| 6.7215 | 0.0012 |
| 7.5205 | 0.1308 |
| 7.5205 | 0.1308 |
| 7.5205 | 0.1308 |
| 9.5314 | 0.0369 |
| 9.5314 | 0.0369 |
| 9.5314 | 0.0369 |
| 10.4965 | 0.1712 |
| 10.4965 | 0.1712 |
| 10.4965 | 0.1712 |

**Table S1.** Excitation energies and the corresponding oscillator strengths of the atomic spectrum in Figure S1.

**Reference**

1 Gaussian 09, Revision D.01, Frisch, M. J.; Trucks, G. W.; Schlegel, H. B.; Scuseria, G. E.; Robb, M. A.; Cheeseman, J. R.; Scalmani, G.; Barone, V.; Mennucci, B.; Petersson, G. A.; Nakatsuji, H.; Caricato, M.; Li, X.; Hratchian, H. P.; Izmaylov, A. F.; Bloino, J.; Zheng, G.; Sonnenberg, J. L.; Hada, M.; Ehara, M.; Toyota, K.; Fu-kuda, R.; Hasegawa, J.; Ishida, M.; Nakajima, T.; Honda, Y.; Kitao, O.; Nakai, H.; Vreven, T.; Montgomery, J. A., Jr.; Peralta, J. E.; Ogli-aro, F.; Bearpark, M.; Heyd, J. J.; Brothers, E.; Kudin, K. N.; Staroverov, V. N.; Kobayashi, R.; Normand, J.; Raghavachari, K.; Rendell, A.; Burant, J. C.; Iyengar, S. S.; Tomasi, J.; Cossi, M.; Rega, N.; Millam, N. J.; Klene, M.; Knox, J. E.; Cross, J. B.; Bakken, V.; Adamo, C.; Jaramillo, J.; Gomperts, R.; Stratmann, R. E.; Yazyev, O.; Austin, A. J.; Cammi, R.; Pomelli, C.; Ochterski, J. W.; Martin, R. L.; Morokuma, K.; Zakrzewski, V. G.; Voth, G. A.; Salvador, P.; Dan-nenberg, J. J.; Dapprich, S.; Daniels, A. D.; Farkas, Ö.; Foresman, J. B.; Ortiz, J. V.; Cioslowski, J.; Fox, D. J. Gaussian, Inc., Wallingford CT, 2009.

2. Legenski, N. *et al.* Force fields for metallic clusters and nanoparticles. *J. Comput. Chem.* **32,** 1711 (2011).

3. Hay, P. J. & Wadt, W. R. Ab initio effective core potentials for molecular calculations. Potentials for the transition metal atoms Sc to Hg. *J. Chem. Phys.* **82,** 270 (1985).
